# The expression of miRNA‐146a‐5p and its mechanism of treating dry eye syndrome

**DOI:** 10.1002/jcla.23571

**Published:** 2020-09-16

**Authors:** Liang Yin, Mingxue Zhang, Tiangeng He, Song Chen

**Affiliations:** ^1^ Department of Ophthalmology Tianjin Medical University General Hospital Tianjin China

**Keywords:** bioinformatics analysis, dry eye syndrome, inflammatory response, IRAK1, microRNA‐146a‐5p

## Abstract

**Objective:**

Dry eye syndrome in which tear fluid quality or abnormality, or kinetic abnormality is caused by various reasons, resulting in decreased tear film stability. In recent years, more and more results from the studies indicate that miRNA alterations are involved in dry eye syndrome. And miRNA‐146a‐5p is a key regulator to regulate the inflammatory response. In this paper, we demonstrated whether miRNA‐146a‐5p could cure dry eye syndrome by regulating target genes based on network analysis.

**Methods:**

In current study, we collected the blood of patients with dry eye disease served as a model group; the blood of healthy people was served as control group. The expression of miRNA‐146a‐5p in the patients was detected by RT‐PCR, the genes controlled by miRNA‐146a‐5p were predicted by TargetScan, miRDB, miRWalk, and PicTar databases, and the genes regulated by miRNA‐146a‐5p which relative with dry eye disease were selected by drawing Venn diagram.

**Results:**

The comparison of the general information between patients and healthy people was no significant difference, and it indicated that the two groups were comparable. The results of databases showed that IRAK1 was one of the target genes regulated by miRNA‐146a‐5p, and it is related to dry eye disease. The expression of miRNA‐146a‐5p was negatively related to IRAK1 mRNA and protein, while IRAK1 had a positive correlation with IL‐6, TNF‐α, and CBP proteins.

**Conclusion:**

These results emphasized that miRNA‐146a‐5p could inhibit the expression of IRAK1, IL‐6, TNF‐α, and CBP to help reduce the inflammatory response in dry eye syndrome.

## Graphical abstract

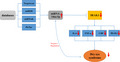

## 1 INTRODUCTION

Dry eye syndrome, also known as keratoconjunctivitis, is a tear and ocular surface disease caused by a variety of factors; the main causes were decreased tear film stability and ocular surface damage induced by tear fluid and quantitative or kinetic abnormalities.^1^ The disease is accompanied by eye dryness, redness, foreign body sensation, and other ocular surface discomfort and visual impairment.^2^ Dry eye syndrome occurs in women over 60 years of age, and its etiology and clinical manifestations vary. The pathogenesis is still unclear. At present, the main clinical application of tear substitutes to relieve dry eye symptoms, but cannot effectively cure dry eye syndrome.^3^ Inflammatory response, apoptosis, and changes in sex hormone levels are all associated with the development of dry eye syndrome, and inflammation is considered to be the most important factor in the pathogenesis of dry eye syndrome.^4^, ^5^

Interleukin‐1 receptor‐associated kinase 1 (IRAK1) is a serine–threonine kinase that mediates Toll‐like receptors (TLR) and IL‐1 signaling pathways. These signaling pathways are critical for regulating immune responses and inflammatory processes.^6^

MicroRNAs (miRNA), a class of non‐coding RNAs, regulated numerous physiological processes via regulating target gene expression Negatively.^7^ Exception expression of microRNA in the dry eye syndrome may be served as a hall marker. Research showed that microRNA‐132 could attenuate LPS‐induced inflammatory injury,^8^ and microRNA‐146a is a key regulator to regulate the inflammatory response.^9^ Therefore, in this paper, we collected the blood of patients with dry eye syndrome served as a model. The blood of healthy people was served as a control group. The results about patients' basic information showed that there was no significant difference and the two groups were comparable. The expression of miRNA‐146a‐5p in model group was decreased compared with control group. The target genes which predicted by TargetScan, miRDB, miRWalk, and PicTar databases proved that IRAK1 is one of the target genes regulated by miRNA‐146a‐5p and the miRNAs which were related with IRAK1 were visualized by cytoscape3.6.1; luciferase reporter assay result showed that miRNA‐146a‐5p could regulate the expression of IRAK1 directly. The results of RT‐PCR showed that miRNA‐146a‐5p was low‐expression in model group compared with control group, and the inhibitor reduced the miRNA‐146a‐5p successfully. The tendency of IRAK1 was negatively expressed with miRNA‐146a‐5p trend. And the amount of TNF‐α, IL‐6, and CBP were positive correlation with IRAK1. At last, western blot was used to detect the relative proteins expression, and the results indicate that miRNA‐146a‐5p could inhibit the expression of IRAK1 protein and the TNF‐α, IL‐6, and CBP proteins were inhibited at the same time. The present study was designed to study the possible mechanism of miRNA‐146a‐3p in dry eye disease and provide new methods for the treatment of dry eye syndrome.

## 2 MATERIALS AND METHODS

### 2.1 The patients

This experiment was carried out in the hospital in China from September 2016 to January 2019. Forty‐three patients with dry eye disease and 53 healthy people were admitted to the study. The necessary information was recorded, and the study was approved by the Institutional Animal Care and Use Committee of Tianjin Medical University General Hospital, and all patients had signed informed consent. The blood of patients with dry eye disease was collected as a model group, and then, 53 healthy people's blood was collected as control group. The expression of miRNA‐146a‐5p was detected by reverse transcriptase‐polymerase chain reaction (RT‐PCR).

### 2.2 Bioinformatics analysis

The target genes of miRNA‐146a‐5p were predicted by TargetScan (TargetScan is a software that specifically analyzes mammalian miRNA target genes. It mainly predicts target genes by searching for conserved 8mer and 7mer sites that match each miRNA seed region. And organized into a database based on the existing analysis results)^10^ (http://www.targetscan.org/vert_72/), miRDB (miRDB is an online database for miRNA target prediction and functional annotations. All the targets in miRDB were predicted by a bioinformatics tool, MirTarget, which was developed by analyzing thousands of miRNA‐target interactions from high‐throughput sequencing experiments. Common features associated with miRNA binding and target downregulation have been identified and used to predict miRNA targets with machine learning methods)^11^ (http://mirdb.org/), miRWalk (the results summarized the information of 13 different miRNA‐mRNA prediction databases. According to different miRNA binding sites: promoter, CDS, 5′ and 3′‐UTR, the mitochondrial genome provides miRNA‐mRNA prediction, which can be based on “Custom Data Set” function to download a custom target list, provides experimentally verified miRNA‐mRNA interactions, and provides links to external databases to collect more information and annotate data)^12^ (http://zmf.umm.uni-heidelberg.de/apps/zmf/mirwalk/), and PicTar (a microRNA target gene for searching animals developed based on the combination of microRNA or microRNA targets. The false positive rate is also low. It was developed by Rajewsky Lab, an expert in the microRNA field)^13^ (https://pictar.mdc-berlin.de/), the results were admitted into draw Venn diagram,^14^ and the results belonged to the four databases were selected. Then, we get the sequence of miRNA‐146a‐5p and the target gene and look for the combining site to identify the relationship between them. Then, we looked for the relative miRNAs of the target gene in dry eye disease and visualized by Cytoscape3.6.1.^15^

### 2.3 Cell culture

The rats were sacrificed and soaked in alcohol, with a volume fraction of 75% for 15 minutes. The rat lacrimal glands were removed with ophthalmic scissors and washed three times with phosphate‐buffered saline (PBS). The upper fascia and surrounding yellow adipose tissue were peeled off, and the dendritic blood vessels and fibrous connective tissue were removed. After rinsing in the original solution, the tissue was cut into 1 ~ 2 mm^3^ pieces with ophthalmic scissors. The prepared two g·L‐1 type II collagenase application solution was added and shaken and digested for 25 minutes at 37°C in an electrothermal oven to obtain a gland cell mass. The culture solution was added to terminate the digestion, and the gland cell pellet was thoroughly blown into a single cell suspension. Filter through a 200 mesh nylon mesh and centrifuged at 800 r/min for 5 minutes. The supernatant was added to a little D‐Hank solution and centrifuged again at 800 r/min. The precipitate was taken, and the culture solution was added and pipetted into a single cell suspension, which was inoculated into a culture flask.^16^

The cells were inoculated into a 50 mL plastic flask at 3 × 10^5^ cm^−2^, and the lacrimal gland epithelial cells were purified by repeated adherence for three times (15 to 20 minutes each), and cultured at 37°C in a volume fraction of 5% CO_2_ incubator. After 36 hours, the first half was changed, and after 48 hours, the whole amount was replaced once. Change the liquid once every 4 days. The first passage was performed after 10 days.

The concentration was 100 μmoL/L H_2_O_2_, and the culture was continued for 60 minutes to induce cell death and recorded as a model group.

### 2.4 Luciferase reporter assay

Luciferase reporter assay was performed to identify the relationship between miRNA‐146a‐5p and IRAK1. In brief, cells at 80% confluence were co‐transfected with wild‐type or mutant IRAK1 3′‐UTR reporters together with miRNA‐146a‐5p or negative control using lipo 2000 (Invitrogen). The plasmid (Promega) encoding luciferase was used to control for transfection efficiency. Cells were lysed 24 hours after transfection and tested for luciferase activities using the Dual‐Luciferase Reporter Assay System (Promega), according to the manufacturer's instructions.

### 2.5 RT‐PCR analysis

The total RNA was isolated from mice cells using TRIzol reagent (Invitrogen) and converted cDNA by OneScript Reverse Transcriptase cDNA Synthesis Kit (TaKaRa). The bulge‐loop TM miRNA reverse primer was used to replace Oligo (dT). 25 μL Dream Taq PCR Master Mix (TaKaRa), 1.5 μL primer (Ribobio), 2 μL cDNA and 20 μL water nuclease‐free in the amplification reaction mixture (50 μL), and the PCR condition were as follows: 95°C (2 minutes, a cycle), 95°C (30 seconds), 58°C (30 seconds), 72°C (1 minutes), 35 cycles in total. Finally, 72°C (10 minutes, a cycle).^17^ GADPH served as the control of APP and U6 snRNA (U6) served as control of miR‐146a‐5p (El Fatimy et al, 2018), the primer sequences were as follows: IRAK1: 5′‐ATCAGGCTTTTTCCCAGGCT‐3′; 5′‐GCACACTATGAGAACTTCCAAGC‐3′; TNF‐α: 5′‐ATAAGAGCAAGGCAGTGGG‐3′; 5′‐TCCAGCAGACTCAATACACA‐3′; IL‐6:5′‐AGCCAGAGTCCTTCAGAGAG‐3′ and 5′‐TCCTTAGCCACTCCTTCTGT‐3′; miRNA‐146a‐5p: 5′‐CTGCCGCTGAGAACTGAATT‐3′; 5′‐CAGAGCAGGGTCCGAGGTA‐3′; GADPH: 5′‐CCATGTTCGTCATGGGTGTGAACCA‐3′; 5′‐GCCAGTAGAGGCAGGGATGATGTTC‐3′; calprotectin: 5′‐CCGGATCCACTAAGCTGGAAGATCACCTGGAGG‐3′; 5′‐CCAAGCTTTACTCTTTGTGGATATCTATGTGGGCTG‐3′.

### 2.6 Western blot

Cells were lysed in radioimmunoprecipitation assay buffer with the Protease Inhibitor Cocktail (Sigma‐Aldrich), separated in sodium dodecyl sulfate‐polyacrylamide gels, and transferred to a polyvinylidene fluoride membrane. The membrane was incubated with anti‐IRAK1, TNF‐α, IL‐6, CBP, and anti‐β‐actin (Abcam) at 4°C overnight, followed by incubation with horseradish peroxidase‐conjugated secondary antibody for 1 hour. Bands were visualized with electrochemiluminescence (ECL).

### 2.7 Statistical analyses

All statistical analyses were performed using SPSS22.0 software. The normality and constant variance for date were tested by Levene's test, and then, data one‐way analysis of variance (ANOVA) followed by the Tukey test was performed to compare with multiple groups. *P* < .05 was considered to be statistically significant. Data are expressed as the mean ± standard error of the mean.

## 3 RESULTS

### 3.1 General information comparison

The patient's basic information was recorded before the patients were admitted into the hospital. The 49 patients with dry eye disease in the hospital were included in the model group, including 32 males and 17 females, and the average age of 37.4 ± 10.8 years. The body's (Body Mass Index, BMI) value was 22.75 ± 3.14, while the control group was 53 healthy subjects who passed the physical examination in the hospital, including 20 males and 33 females, the average age was 38.2 ± 10.7, body BMI is also within the normal range. There was no statistical difference between the general information of the model combination control group, and it was comparable. See Table 1 for details.

**TABLE 1:** Comparison of general conditions between the two groups of subjects

Project	Model group	Control group
Number	49	53
Male/female	32/17	20/33
Age (x ± s), year	57.4 ± 10.8	58.2 ± 10.7
BMI (x ± s), kg/m^2^	22.75 ± 3.14	24.32 ± 1.57

### 3.2 MiRNA‐146a‐5p was low‐expression in the patients with dry eye disease

**FIGURE 1:**
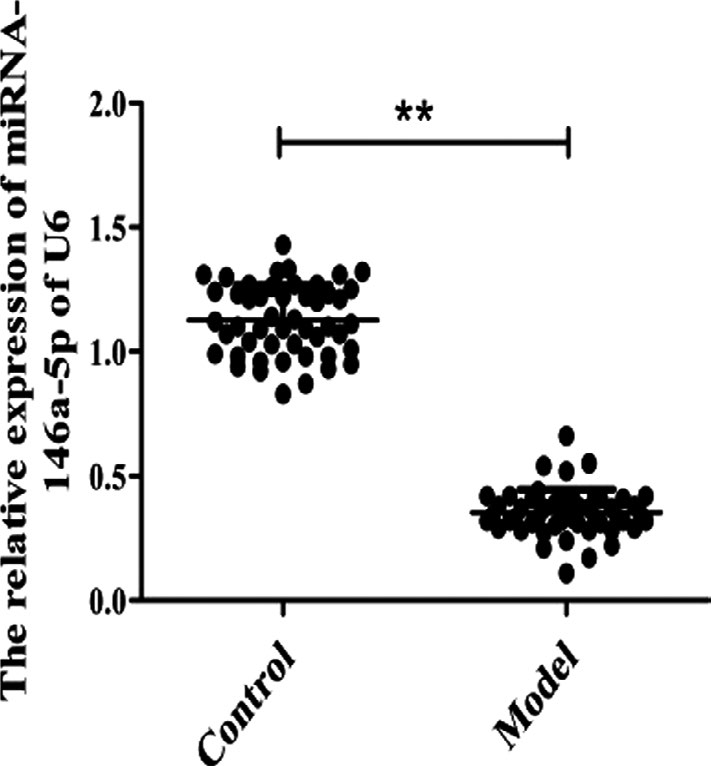
The relative miRNA‐146a‐5p expression in dry eye syndrome. ^**^ *P* < .001 vs Control group

To explore the expression of miRNA‐146‐5p in the patients with dry eye disease, the blood of the model and control group was selected, and the expression of miRNA‐146a‐5p was detected by RT‐PCR. The result showed that miRNA‐146a‐5p was low expression in model group compared with the control group. The difference between the two groups was statistically significant (Figure 1). It indicated that miRNA‐146a‐5p may be the marker of dry eye disease.

### 3.3 IRAK1 is the target gene regulated by miRNA‐146a‐5p

**FIGURE 2:**
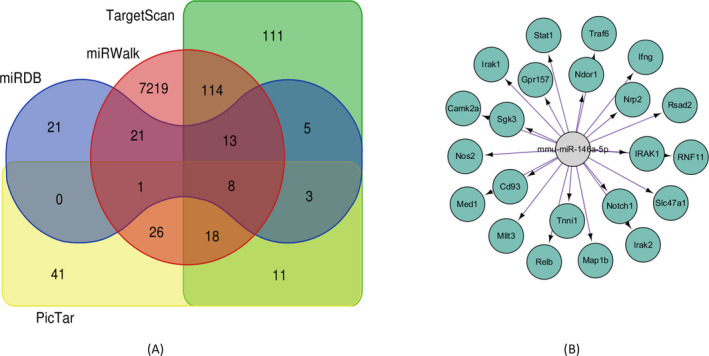
IRAK1 is the target gene regulated by miRNA146a‐5p in dry eye syndrome. A, The target genes regulated by miRNA‐146a‐5p were detected by databases; B, the IRAK1 which was the miRNA‐146a‐5p target gene was visualized by cytoscape

To predict the target genes of miRNA‐146a‐5p, the databases were used. We can know that there are seven genes regulated by miRNA‐146a‐5p determined by miRDB, miRWalk, PicTar, TargetScan databases (Figure 2A), including IRAK1, TRAF‐6, NOVA1, and so on. Among them, IRAK1 is related to inflammatory response, while inflammatory response would lead to dry eye disease. Therefore, we will focus on the specific mechanism of dry eye disease with miRNA‐146a‐5p and IRAK1 and explore the potential mechanism.

We explore the targeting relationship between miRNA‐146a‐5p and IRAK1 by establishing the network using cytoscape3.6.1. The network showed that there are numerous genes regulated by miRNA‐146a‐5p, and IRAK1 was one of them (Figure 2B).

### 3.4 IRAK1 is the target gene regulated by miRNA‐146a‐5p

**FIGURE 3:**
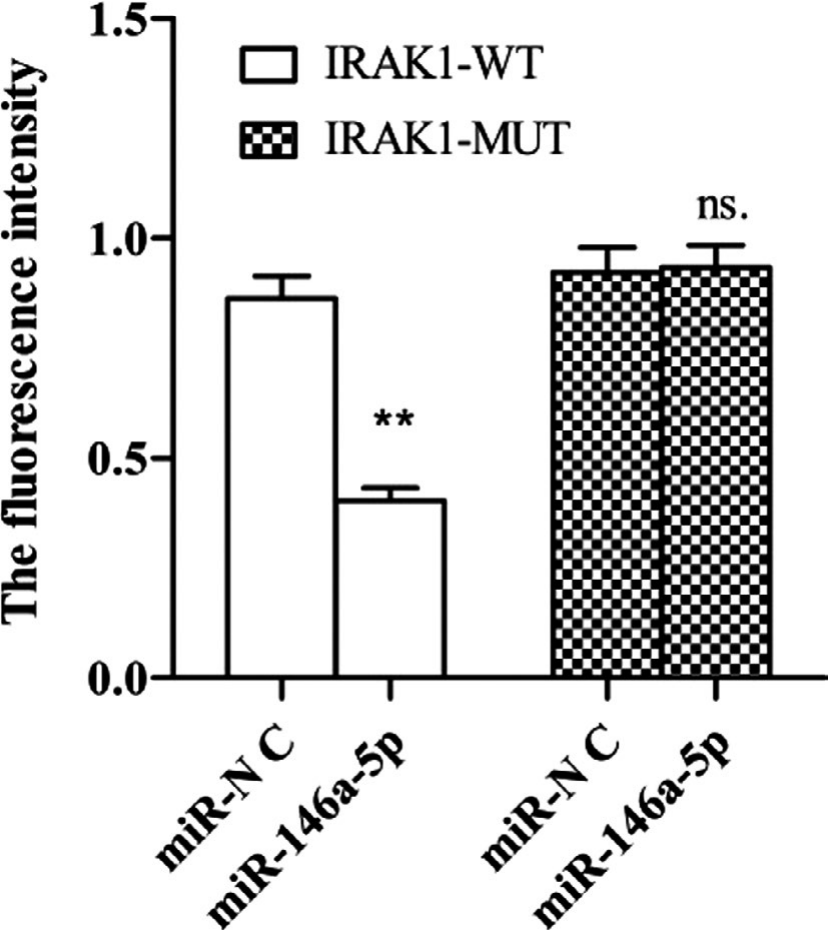
The fluorescence intensity was measured by luciferase report assay. The overexpressed miR‐146a‐5p was able to bind to IRAK1‐WT, the fluorescence intensity was weakened, and the difference was significant, *P* < .01. In combination with IRAK1‐MUT, there is no difference in fluorescence intensity

To determine whether the inhibition of IRAK1 by miR‐146a‐5p occurred via these predicted miR‐146a‐5p binding sites, the combining site was mutated. Luciferase reporter assays indicated that the IRAK1 mutant 3ʹ‐UTR interrupted miR‐146a‐5p‐mediated repression, and it noted that miRNA‐146a‐5p could regulate the expression of IRAK1 directly (Figure 3).

### 3.5 MiRNA‐146a‐5p inhibits inflammatory release by targeting IRAK1 mRNA

**FIGURE 4:**
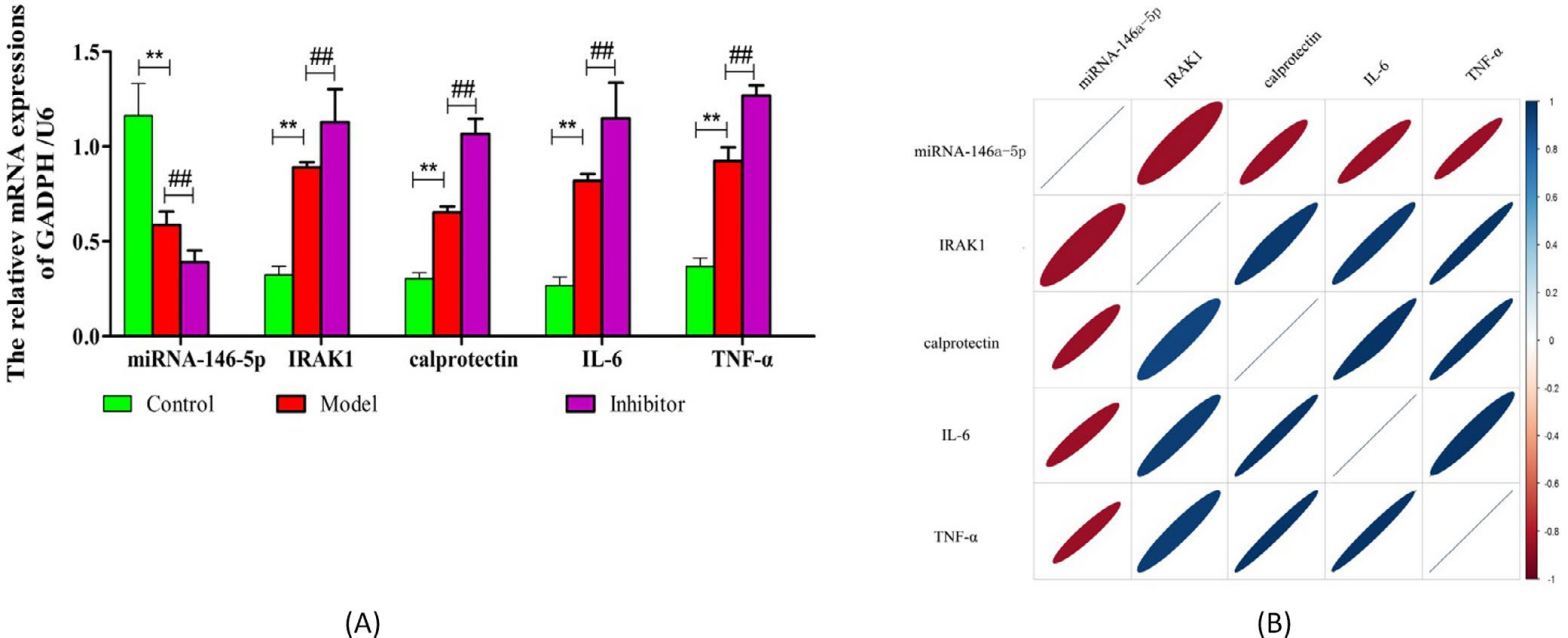
The expression of miRNA‐146a‐5p and IRAK1. A, The expression of miRNA‐146a‐5p, IRAK1, calprotectin, IL‐6, and TNF‐α was analyzed by RT‐PCR; the expression levels were semi‐quantified by densitometric measurements, normalized with U6/GADPH; ^**^ *P* < .01 vs CON; ^##^ *P* < .01 vs Model; B, the relationship of miRNA‐146a‐5p, IRAK1, calprotectin, IL‐6, and TNF‐α mRNA. Red and blue mean the negative correlation/positive correlation, respectively

Then, we determined the expression of IRAK1 when miRNA‐146a‐5p with inhibition treatment (Figure 4A). We found that compared with the control group, the expression of miRNA‐146a‐5p was decreased, and IRAK1 increased at the same time in the model group. While the inhibitor group showed that the inhibition of miRNA‐146a‐5p would lead to an increase of IRAK1 mRNA, and when the expression of miRNA‐146a‐5p raising, the IRAK1 mRNA would decrease at the same time. The relationship between miRNA‐146a‐5p and IRAK1 was negatively correlated. These results suggested that the highly conserved sequence of the IRAK1 3ʹ‐UTR is the primary miR‐146a‐5p binding site and further confirmed that miR‐146a‐5p directly inhibits IRAK1 (Figure 4B).

Then, we detected the expression of IL‐6, calprotectin, and TNF‐α (Figure 4A), and the result showed that the expressions of IL‐6, calprotectin and TNF‐α were increased in model group compared with control group, and the expressions of IL‐6, calprotectin and TNF‐α in inhibitor group were decreased obviously (Figure 4B). It indicated that the upregulating miRNA‐146a‐5p and targeting IRAK1 mRNA may inhibit inflammatory release.

At last, we detected the relationship of miRNA‐146a‐5p, IRAK1, calprotectin, IL‐6, and TNF‐α mRNA, and the result showed that miRNA‐146a‐5p was negatively related with IRAK1,while the expression of IL‐6, calprotectin, and TNF‐α had a positive relation with IRAK1 and negatively related with miRNA‐146a‐5p (Figure 4C).

### 3.6 MiRNA‐146a‐5p attenuates inflammatory response by regulating TRAF‐6 protein

**FIGURE 5:**
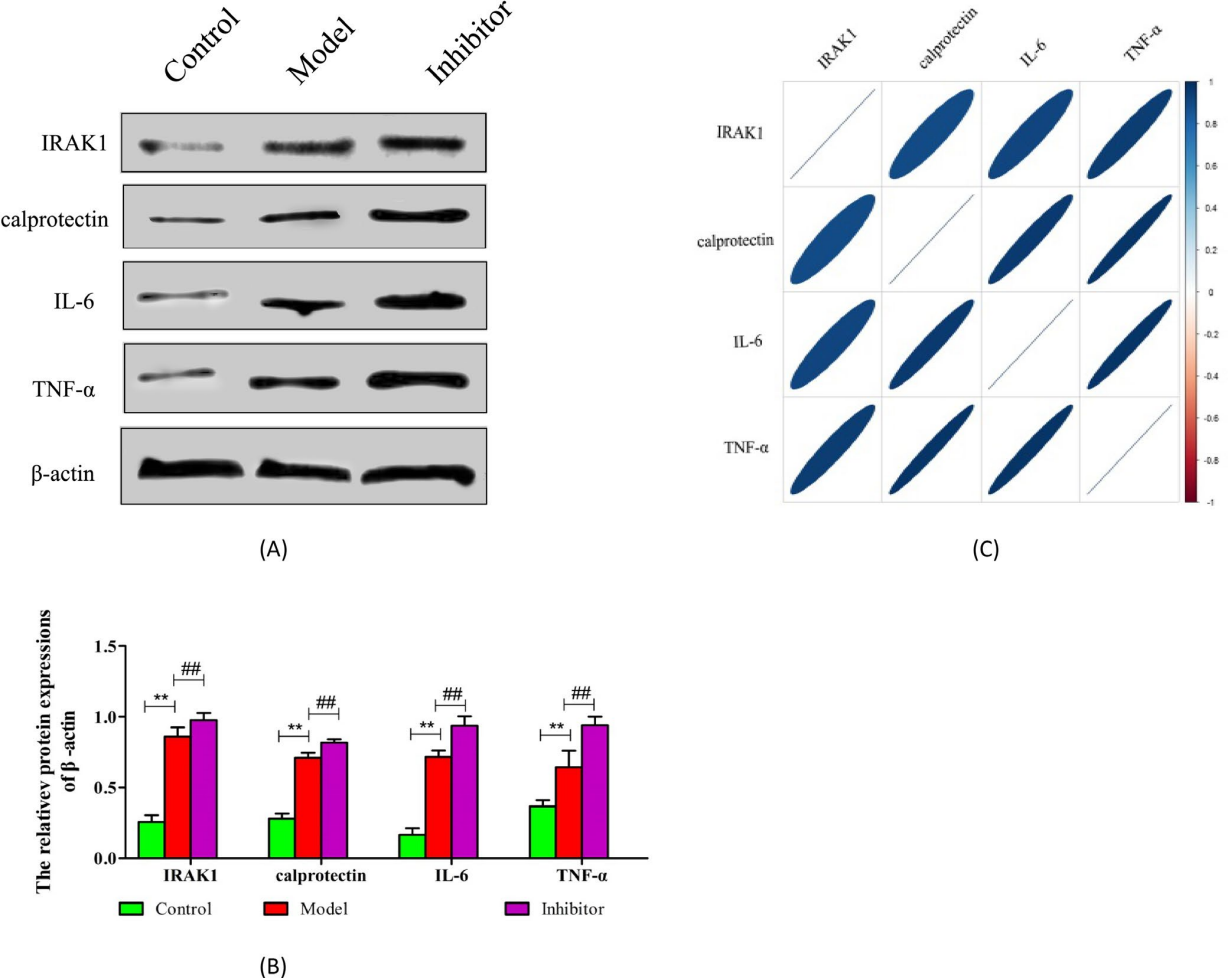
The expression of IRAK1, calprotectin, IL‐6, and TNF‐α proteins. A, The expression of IRAK1, IL‐6, calprotectin, and TNF‐α proteins was determined by Western blot; B, the expression levels were semi‐quantified by densitometric measurements, normalized with β‐actin. n = 3, ^**^ *P* < .01 vs CON; ^##^ *P* < .01 vs Model; C, the relationship of TRAF6, IL‐6, calprotectin, and TNF‐α proteins. Red means the negative correlation; blue means positive correlation

In order to prove the effects of miRNA‐146a‐5p on the expression of IRAK1 protein, Western Blot was used. The IRAK1 protein was significantly increased in model group compared with control group. While the expression of IRAK1 protein was the highest in the inhibitor group (Figure 5A), the difference was statistically significant (*P* < .01). MiRNA‐146a‐5p inhibitor attenuates the inhibitory effect of miR‐146a‐5p on IRAK1 protein expression.

At last, we detected the relationship of IRAK1, IL‐6, and TNF‐α proteins, and the result showed that miRNA‐146a‐5p was negatively related with IRAK1, while the expression of calprotectin, IL‐6, and TNF‐α proteins had a positive relation with IRAK1 (Figure 5B).

## 4 DISCUSSION

Dry eye syndrome refers to a general term for a type of disease in which the composition and volume of tears or abnormalities in kinetics cause instability of the tear film, accompanied by eye discomfort such as dryness,^18^ visual fatigue,^19^ photophobia,^20^ and decreased vision.^21^ In terms of classification, it is a kind of chalk disease; that is, there is no abnormality in appearance and the patient has obvious eye discomfort.

Dry eye syndrome is usually accompanied by inflammation.^22^ Under the action of the body's immune system, a certain number of lymphocytes infiltrate the ocular surface tissue and lacrimal gland, releasing a large number of inflammatory factors, causing the appearance of inflammation in the body, further affecting the tear secretion system of the human body, leading to pathological changes in the amount and composition of tear secretion.^23^ In order to escape the attack from the lacrimal immune system, these lymphocytes secrete inflammatory factors, which also have the effect of affecting the secretion of normal glands; in addition, inflammatory factors can also exert effects on sympathetic and parasympathetic nerves, so that the sensory nerve function corresponding to the ocular surface decline, eventually destroying the integrity of the tear film.^24^

Now, more and more researches pay attention to miRNA in various diseases. Among them, the microRNA related to dry eye syndrome is a hot topic, too.^25^ And it could regulate numerous physiological processes via binding to the 3′ untranslatable region (3′‐UTR) of their mRNA targets.^26^ Up to now, many study have demonstrated that miRNA‐146a is an important regulator of the inflammatory response,^27^ so its regulation of inflammatory response has an important impact on dry eye syndrome. However, whether miRNA‐146a‐5p could cure dry eye syndrome and its specific mechanism remains is still unclear. Therefore, **i**n this paper, we collected the blood of patients with dry eye disease served as a model group; the blood of healthy people was served as a control group. We found that the comparison of the general information between patients and healthy people was no significant difference, and it indicated that the two groups were comparable. The expression of miRNA‐146a‐5p was lower in model group compared with that in control group. The target genes regulated by miRNA‐146a‐5p were predicted by TargetScan, miRDB, miRWalk, and PicTar databases, and the genes regulated by miRNA‐146a‐5p which relative with dry eye disease were selected by draw Venn diagram. The results of databases showed that IRAK1 was one of the target genes regulated by miRNA‐146a‐5p and it is related to dry eye disease. The results of luciferase reporter assay showed that miRNA‐146a‐5p could regulate the expression of IRAK1 directly. The expression of miRNA‐146a‐5p was negatively related to IRAK1 mRNA and protein, while IRAK1 had a positive correlation with IL‐6, TNF‐α, and CBP proteins.

## 5 CONCLUSIONS

These results emphasized that the up‐regulation of miRNA‐146a‐5p could inhibit the expression of IRAK1, IL‐6, TNF‐α, and CBP to help reduce the inflammatory response in dry eye syndrome.

## CONFLICT OF INTEREST

The authors declared no conflict of interest.
